# Colonic thickening on computed tomography—does it correlate with endoscopic findings? A protocol for systematic review

**DOI:** 10.1186/s13643-016-0381-7

**Published:** 2016-12-13

**Authors:** Subashini Chandrapalan, Faraz Tahir, Rakesh Sinha, Ramesh Arasaradnam

**Affiliations:** 1 University Hospital of North Midlands, Stoke-on-Trent, UK; 2 South Warwickshire NHS Foundation Trust, Warwick, UK; 3 University Hospital of Coventry and Warwickshire, England, UK

**Keywords:** Computed tomography scan, Colon, Mural thickening, Endoscopy

## Abstract

**Background:**

Colonic mural thickening is often a finding in standard computed tomography (CT) scans of the abdomen. It often presents clinician with a dilemma on when a further endoscopic evaluation is needed, especially in the absence of guidelines. The aim of this systematic review is to evaluate the significance of bowel wall thickening and to assess its correlation with endoscopy.

**Methods:**

This systematic review will be reported in accordance with the preferred reporting items for systematic review and meta-analysis protocols (PRISMA-P) 2015 statement. The search strategy will initially be developed in MEDLINE and adapted for use in EMBASE, MEDLINE, NHS evidence and TRIP. Two reviewers will independently conduct a study selection, data extraction and risk of bias assessment for the screened studies.

Data synthesis will be conducted using Review Manager software 5.3. The outcome of any dichotomous data will be presented as relative risk with confidence intervals.

**Discussion:**

It is extremely useful for the practising clinician to know which patients need further endoscopic evaluation. Even though there are several studies on this issue, none of them have attempted to produce a systematic review. We hope this systematic review will provide a substantiate evidence for future clinical practice.

**Systematic review registration:**

PROSPERO CRD42016039378

**Electronic supplementary material:**

The online version of this article (doi:10.1186/s13643-016-0381-7) contains supplementary material, which is available to authorized users.

## Background

CT scanning is a widely used imaging modality in the diagnostic work up of bowel pathologies. Recent advances in CT scanning, such as the multi-detector technology allows higher accuracy and sensitivity in the diagnosis of abdominal pathology.

One of the findings on standard abdominal CT imaging, potentially indicating pathology, is that of colonic mural thickening (MT). Colonic wall thickening may be a reflection of inflammatory, infective, ischaemic and neoplastic pathologies [1–3]. On the other hand, MT may simply be due to benign strictures or collapsed segments of the colon [4, 5].

In the setting of abnormal MT, patients may have to undergo lower GI endoscopy for further examinations. However, currently there are no definitive guidelines to suggest when an endoscopic evaluation is needed [6, 7]. This often results in a diagnostic dilemma, especially when a clinical index of suspicion is low.

Several studies have evaluated the clinical significance of MT on CT imaging and its correlation with subsequent endoscopic findings. Few studies have illustrated the differentiating features of benign from malignant MT. In this study, we will conduct a systematic review of MT on abdominal CT imaging and its correlation with colonic findings, in order to provide a more precise and reliable evidence for the significance of MT identified on abdominal CT [8].

### Objectives

The aim of this systematic review is to evaluate the colonic mural thickening on CT and its correlation with pathologies identified by endoscopy within a month.

## Methods

In accordance with the guidelines, our systematic review protocol has been registered with the international prospective register of systematic reviews (PROSPERO) and it is reported according to the recommendations from the preferred reporting items for systematic review and meta-analysis protocols (PRISMA-P) 2015 statement [9] (Additional file 1).

### Study eligibility

Studies will be selected according to the criteria outlined below.

### Types of study designs

All of the prospective and retrospective comparative cohort studies, case controlled, nested case-control studies and cross sectional studies will be included. We will exclude review articles, editorials, consensus statements and opinions. Review articles will be examined for identification of original studies.

### Types of participants

We will include studies examining general adult human population of 18 years or older. This will include both healthy people and those with colonic pathologies.

### Types of intervention

We will include studies which examine people who have had endoscopic colonic evaluation following a CT scan. The studies describe non-colonic thickening, i.e. sites other than from ileocaecal valve to rectosigmoid junction will be excluded.

### Language and length of time

Studies which are published in English and available as full texts in the medical database with no time limit, will be included.

### Types of objectives

#### Primary outcome

- Probability of endoscopically identified bowel pathology in those with colonic mural thickening on CT.

#### Secondary outcome

- Significance of colonic thickening at different sites of the colon.

### Search strategy and identification of studies

Literature search strategies will be developed by two librarians who are experts in literature searches using medical subject headings (MeSH) and text words related to the title. We will search Medline, EMBASE, NHS evidence and TRIP using various combinations of keywords and subject headings for the articles related to our title.

The following search strategy, developed in MEDLINE (Ovid) using various refined search terms to have a wider coverage as possible, will be adapted to the syntax and subject headings of the other databases.

1. ((colon* OR mucosal OR bowel OR sigmoid) ADJ2 thick*).ti,ab;
2. exp TOMOGRAPHY, X-RAY COMPUTED/;
3. ((comput* ADJ2 tomograph*) OR "CT scan" OR "X-ray CT" OR "CAT scan").ti,ab;
4. 2 OR 3;
5. exp COLONOSCOPY/;
6. colonoscop*.ti,ab;
7. sigmoidoscop*.ti,ab;
8. ENDOSCOPY/;
9. endoscop*.ti,ab;
10. 5 OR 6 OR 7 OR 8 OR 9;
11. 1 AND 4 AND 10;
12. 11 [Limit to: (Language English) and Humans];

To ensure maximum capture, we will scan the reference lists of included studies or relevant reviews identified through the search for the potential studies.

### Study selection

We will carry out a two-stage processes.

- Stage 1: the titles and abstracts of the identified studies will be scanned by two reviewers for relevance to the topic. The studies which are identified as non-relevant will be excluded. The studies which fall between the borderline will be passed to the team for further review.
- Stage 2: full text materials of all the relevant articles will be reviewed by two independent reviewers using Scottish Intercollegiate Guideline Network (SIGN) checklist, and a final selection will be made.

The PRISMA flow chart will be produced to ensure transparency.

### Data extraction and management

Data will be extracted from all the included studies using a data extraction form (Additional file 2). This is a Microsoft Word document, and the extracted data will be entered electronically to facilitate the summary and analysis. The following data will be extracted: (1) study details (study year, study design, study aim); (2) study and population characteristics (sample size, inclusion/exclusion criteria, study mean age, gender distribution; (3) intervention (number of patients who had endoscopy); (4) outcomes (primary and secondary outcome of study), (5) results (outcome of endoscopy, site of abnormality, type of abnormality). Where needed, further calculations will be made from available data and the authors will be contacted if necessary.

Data extraction will be done by two reviewers independently, to increase the accuracy, and any disagreements will be resolved by consensus or by involving a third reviewer.

### Risk of bias assessment

The risk of bias will be assessed using SIGN checklists for methodology assessment. These look into study characteristics, population characteristics, statistical analysis and study outcomes. Each of these domains will be offered a score, and the overall score will determine the study quality (high, acceptable and poor). This will be done by one reviewer and will be double-checked by the other [10]. Any disagreement will be resolved by discussion. These tools are included in Additional file 3.

### Data synthesis and analysis

Data analysis will be conducted using latest the version of Review Manager software 5.3. The data will be presented in texts and tables to provide a descriptive summary and explanation of the characteristics of the included studies. If the heterogeneity is too high, the results will be narratively synthesized. Rigorous and transparent approach will be adopted to minimise the potential for bias.

### Effect measures for dichotomous data

The outcome measures for dichotomous data (probability of people with bowel pathology on endoscopy among those with MT on CT, probability of people who have bowel pathology at different sites) will be reported as relative risk with 95% confidence intervals.

### Effect measures for continuous data

Continuous outcomes will be summarised as means and will be presented using standard error or standard deviation. The amount by which the intervention changes the outcome will be presented as a mean difference with 95% confidence intervals.

We will assess the heterogeneity of the summary effect results and studies which are similar in design, participants and comparators will be pooled together using a random effect meta-analysis model. The heterogeneity of the studies will be examined from forest plot and chi-square tests [11].

Publication bias will be addressed by visually examining funnel plots if more than ten studies were identified. If sufficient studies are available, we will perform subgroup analysis for different age groups, sex, CT parameter techniques, reporter of the CT and sites of colonic mural thickening.

## Discussion

Although several studies examined the significance of colonic thickening and the correlation with follow-up endoscopy, no systematic review has been produced so far. To our knowledge, this is the first systematic review examining the issue. We hope this review will produce a high-quality evidence for future practice and will inform the future trials.

## Additional files

Additional file 1: PRISMA-P (Preferred Reporting Items for Systematic review and Meta-Analysis Protocols) 2015 checklist: recommended items to address in a systematic review protocol*. (DOC 84.5 kb)
Additional file 2: Data Extraction Form. (DOCX 28.8 kb)
Additional file 3: Methodology check list for cohort studies. (DOCX 30.7 kb)

## Abbreviations

CT: Computed tomography
MeSH: Medical subject headings
MT: Mural thickening
PRISMA: Preferred reporting items for systematic reviews and meta-analysis
PRISMA-P: Preferred reporting items for systematic reviews and meta-analysis for protocols
PROSPERO: International prospective register of systematic reviews
SIGN: Scottish Intercollegiate Guideline Network
