# Supplementary material for: Colonic thickening on computed tomography—does it correlate with endoscopic findings? A protocol for systematic review
Source: Syst Rev. 2016 Dec 13;5:213. doi: 10.1186/s13643-016-0381-7 (PMC5155394; doi:10.1186/s13643-016-0381-7)
Supplement: Additional file 2: — Data Extraction Form. (DOCX 28.8 kb) [file 13643_2016_381_MOESM2_ESM.docx]

**Data Extraction Form**

| **Study details** | |
| --- | --- |
| Surname of the first author |  |
| Year of publication |  |
| Study design |  |
| No. of study centres |  |
| Aim of the study |  |
| **Study population** | |
| Sample size |  |
| Inclusion criteria |  |
| Exclusion criteria |  |
| **Population characteristics** | |
| Sample mean age |  |
| Sex distribution (No.) | M : F |
| Ethnicity |  |
| **Intervention** | |
| No of patients who had endoscopy |  |
| No of patients who didn’t have endoscopy |  |
| **Outcomes (List)** | |
| Primary outcomes |  |
| Secondary outcomes |  |
| **Authors conclusion (Free Text)** | |

| **Study results** | | |
| --- | --- | --- |
|  | No of Abnormal endoscopy/Total no of patients | No of Normal endoscopy/Total no of patients |
| Outcome of endoscopy |  |  |

| **Site of abnormality** | | |
| --- | --- | --- |
|  | At the site of BWT on CT | Away from BWT on CT |
| Total No of patients |  |  |
| BWT of transverse colon |  |  |
| BWT of Ascending colon |  |  |
| BWT of Descending colon |  |  |
| BWT of sigmoid colon |  |  |
| **Type of abnormality** | | |
| Malignant |  |  |
| Inflammatory |  |  |
| Benign |  |  |
|  |  |  |

**Comments**
