# Supplementary material for: Colonic thickening on computed tomography—does it correlate with endoscopic findings? A protocol for systematic review
Source: Syst Rev. 2016 Dec 13;5:213. doi: 10.1186/s13643-016-0381-7 (PMC5155394; doi:10.1186/s13643-016-0381-7)
Supplement: Additional file 3: — Methodology check list for cohort studies. (DOCX 30.7 kb) [file 13643_2016_381_MOESM3_ESM.docx]

| **Domain** | **Yes** | **No** | **Can’t say** | **Doesn’t apply** |
| --- | --- | --- | --- | --- |
| 1. **Internal validity** | | | | |
| The study addresses an appropriate and clearly focused question |  |  |  |  |
| **Selection of Subjects** | | | | |
| The two groups being studied are selected from source populations that are comparable in all respects other than the factor under investigation |  |  |  |  |
| The study indicates how many of the people asked to take part did so, in each of the groups being studied |  |  |  |  |
| The likelihood that some eligible subjects might have the outcome at the time of enrolment is assessed and taken into account in the analysis |  |  |  |  |
| What percentage of individuals or clusters recruited into each arm of the study dropped out before the study was completed |  | | | |
| Comparison is made between full participants and those lost to follow up, by exposure status |  |  |  |  |
| **Assessment** | | | | |
| The outcome is clearly defined |  |  |  |  |
| The assessment of outcome is made blind to exposure status. If the study is retrospective this may not be applicable |  |  |  |  |
| Where blinding was not possible, there is some recognition that knowledge of exposure status could have influenced the assessment of outcome |  |  |  |  |
| The method of assessment of exposure is reliable |  |  |  |  |
| Evidence from other sources is used to demonstrate that the method of outcome assessment is valid and reliable |  |  |  |  |
| Exposure level or prognostic factor is assessed more than once |  |  |  |  |
| **Confounding** | | | | |
| The main potential confounders are identified and taken into account in the design and analysis |  |  |  |  |
| **Statistical analysis** | | | | |
| Have confidence intervals been provided |  |  |  |  |
| **Overall assessment of the study** | | | | |
| Taking into account clinical considerations, your evaluation of the methodology used, and the statistical power of the study, do you think there is clear evidence of an association between exposure and outcome |  |  |  |  |
| How well was the study done to minimise the risk of bias or confounding | H. Quality Acceptable Unaccept | | | |
| Are the results of the study directly applicable to the patient group targeted in this guidelines |  |  |  |  |

**Methodology check list for case-control studies**

| **Domain** | **Yes** | **No** | **Can’t say** | **Doesn’t apply** |
| --- | --- | --- | --- | --- |
| 1. **Internal validity** | | | | |
| The study addresses an appropriate and clearly focused question |  |  |  |  |
| **Selection of subjects** | | | | |
| The cases and controls are taken from comparable populations |  |  |  |  |
| The same exclusion criteria are used for both cases and controls |  |  |  |  |
| What percentage of each group (cases and controls) participated in the study | Cases: Controls: | | | |
| Comparison is make between participants and non-participants to establish their similarities or differences |  |  |  |  |
| Cases are clearly defined and differentiated from controls |  |  |  |  |
| It is clearly established that controls are non-cases |  |  |  |  |
| **Assessment** | | | | |
| Measures will have been taken to prevent knowledge of primary exposure influencing case ascertainment |  |  |  |  |
| Exposure status is measured in a standard, valid and reliable way |  |  |  |  |
| **Confounding** | | | | |
| The main potential confounders are identified and taken into account in the design and analysis |  |  |  |  |
| **Statistical analysis** | | | | |
| Confidence intervals are provided |  |  |  |  |
| 1. **Overall assessment of the study** | | | | |
| How well was the study done to minimise the risk of bias or confounding | H. Quality Acceptable Reject | | | |
| Taking into account clinical considerations, your evaluation of the methodology used, and the statistical power of the study, do you think there is clear evidence o an association between exposure and outcome? |  |  |  |  |
| Are the results of this study directly applicable to the patient group targeted by this guideline? |  |  |  |  |
